# Complete female mitochondrial genome of *Mytilus chilensis*

**DOI:** 10.1080/23802359.2017.1289343

**Published:** 2017-02-16

**Authors:** Beata Śmietanka, Artur Burzyński

**Affiliations:** Institute of Oceanology, Polish Academy of Sciences, Sopot, Poland

**Keywords:** DUI, mitogenomics, South American mussels, East Pacific

## Abstract

The controversy surrounding the origin of antitropical distribution of *Mytilus* mussels and the taxonomic status of southern hemisphere populations remain unsolved, despite the efforts. One of the limiting factors remains the lack of the complete sequences of the representative mitochondrial genomes which would allow their proper comparison with the relatively well-represented northern hemisphere congeneric mussels. To fill this gap we sequenced the representative maternal (F) genome of a native Chilean mussel. The genome is 16,748bp long and structurally identical to the northern hemisphere *M. edulis* and *M. galloprovincialis* F genomes. However, the genetic distance from them (≈5%) is twice as high as the maximum distance between them (<2.5%). Thus, the notion that the name *M. chilensis* should be used for native Chilean *Mytilus* mussels, with the same rank as *M. galloprovincialis* and *M. edulis* is supported.

The *Mytilus edulis* species complex consists of three well-recognized marine mussels: *M. edulis*, *M. trossulus, M. galloprovincialis* and several populations found on the southern hemisphere (Hilbish et al. 2000). These southern populations have mixed ancestry, some are recently introduced by humans and some are native, with different distances from *M. edulis* and *M. galloprovincialis* of the northern hemisphere (Borsa et al. 2007). It has been shown that three separate mitochondrial lineages exist in southern hemisphere *Mytilus* spp. (Gérard et al. 2008). The status of these populations remains controversial. Some researchers call them either *M. edulis* or *M. galloprovincialis* of southern hemisphere (Westfall & Gardner 2010), while others use the term *M. chilensis* (Oyarzún et al. 2016). The officially accepted classification gives the native South American *Mytilus* mussels subspecies rank and the name *M. edulis platensis* d'Orbigny, 1842 and is supported by a critical review of literature (Borsa et al. 2012). It has been speculated that as many as four *Mytilus* species are present in the Magellane region of Chile (Oyarzún et al. 2016). Unfortunately, these are based on a limited number of nuclear and low resolution mitochondrial markers. Clearly, there is a need for better markers. Complete mitochondrial sequences contain enough information to precisely date the evolutionary events within the *M. edulis* species complex (Śmietanka et al. 2010), making them excellent marker candidates.

All *Mytilus* mussels exhibit an unusual system of mitochondrial DNA inheritance (Skibinski et al. 1994; Zouros et al. 1994). In this system two divergent mitochondrial genomes exist. However, the paternally transmitted M genome is present only in males whereas the maternally inherited F genome is more ubiquitous. Therefore, the F genome is more suitable for marker development.

Here we announce the complete sequence of the F mitochondrial genome from South American *M. chilensis*. It was isolated from a female specimen of Chilean *Mytilus* mussel sampled near Niebla, Valdivia (39° 51' 20” S, 73° 23' 35” W). It is representative for the local native population. The *16S* sequence of this genome perfectly match native haplotypes (AM904582 and AM904585 from Gérard et al. 2008). The complete sequence was obtained in two steps: long range PCR, followed by re-amplifications and direct sequencing of shorter PCR products, as described previously (Śmietanka et al. 2010). The sequence has been deposited in GenBank under accession number KT966847 and the specimen is stored under CH1 accession number in our repository.

The number and order of genes in this genome is the same as in all other published mitochondrial F genomes from members of *M. edulis* species complex. There are also no major differences within the Control Region. The phylogenetic tree based on genetic distances between the newly sequenced genome and its closest published relatives was constructed (Figure 1). The analysis confirms distinctiveness of southern hemisphere American *Mytilus* F genomes, supporting the view that *M. chilensis* should have the same taxonomic rank as *M. galloprovincialis* and *M. edulis* and should not be referred to as “southern hemisphere *M. galloprovincialis”* or “southern hemisphere *M. edulis*”.

**Figure 1.**
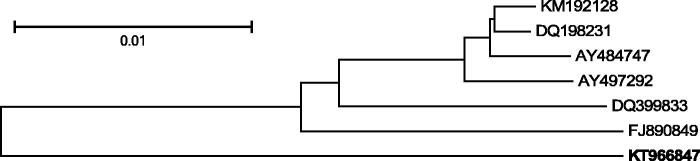
Comparative analysis of the announced mitogenome. The following six complete F mitochondrial genomes, most similar (more than 94% sequence similarity) to the announced F genome of *M. chilensis* (KT966847, in bold) have been downloaded from GenBank: KM192128 and DQ198231 *M. trossulus* from the Baltic Sea (Zbawicka et al. 2007, 2014), AY484747 *M. edulis* from the Atlantic (Boore et al. 2004), AY497292 *M. galloprovincialis* from the Atlantic (Mizi et al. 2005), DQ399833 *M. galloprovincialis* from the Black Sea (Venetis et al. 2007), FJ890849 *M. galloprovincialis* from the Mediterranean Sea (Burzyński & Smietanka 2009). The sequences were aligned using MUSCLE (Edgar 2004). All alignment positions containing gaps and missing data were eliminated. There were a total of 16,247 positions in the final data set. The tree was inferred using the Neighbour-Joining method (Saitou & Nei 1987). The distances between sequences were computed as the number of base differences per site. The tree is drawn to scale, with branch lengths in the same units. The optimal tree, with the sum of branch lengths = 0.08 is shown. Bootstrap support (1000 replicates, Felsenstein 1985) was greater than 95% for all bipartitions. All analyses were conducted in MEGA6 (Tamura et al. 2013).
